# Evidence for exposure dependent carriage of malaria parasites across the dry season: modelling analysis of longitudinal data

**DOI:** 10.1186/s12936-023-04461-1

**Published:** 2023-02-03

**Authors:** Eva Stadler, Deborah Cromer, Samson Ogunlade, Aissata Ongoiba, Safiatou Doumbo, Kassoum Kayentao, Boubacar Traore, Peter D. Crompton, Silvia Portugal, Miles P. Davenport, David S. Khoury

**Affiliations:** 1 The Kirby Institute, UNSW Sydney, Sydney, NSW 2052 Australia; 2 Malaria Research and Training Centre, Department of Epidemiology of Parasitic Diseases, International Center of Excellence in Research, University of Sciences, Technique, and Technology of Bamako, 91094 Bamako, Mali; 3 Malaria Infection Biology and Immunity Section, Laboratory of Immunogenetics, National Institute of Allergy and Infectious Diseases, National Institutes of Health, Rockville, USA

**Keywords:** Seasonal transmission, *Plasmodium falciparum*, Within-host model, Parasite carriage, Mali

## Abstract

**Background:**

In malaria endemic regions, transmission of *Plasmodium falciparum* parasites is often seasonal with very low transmission during the dry season and high transmission in the wet season. Parasites survive the dry season within some individuals who experience prolonged carriage of parasites and are thought to ‘seed’ infection in the next transmission season.

**Methods:**

Dry season carriers and their role in the subsequent transmission season are characterized using a combination of mathematical simulations and data analysis of previously described data from a longitudinal study in Mali of individuals aged 3 months–12 years (n = 579).

**Results:**

Simulating the life-history of individuals experiencing repeated exposure to infection predicts that dry season carriage is more likely in the oldest, most exposed and most immune individuals. This hypothesis is supported by the data from Mali, which shows that carriers are significantly older, experience a higher biting rate at the beginning of the transmission season and develop clinical malaria later than non-carriers. Further, since the most exposed individuals in a community are most likely to be dry season carriers, this is predicted to enable a more than twofold faster spread of parasites into the mosquito population at the start of the subsequent wet season.

**Conclusions:**

Carriage of malaria parasites over the months-long dry season in Mali is most likely in the older, more exposed and more immune children. These children may act as super-spreaders facilitating the fast spread of parasites at the beginning of the next transmission season.

**Supplementary Information:**

The online version contains supplementary material available at 10.1186/s12936-023-04461-1.

## Background

Malaria caused an estimated 627,000 deaths worldwide in 2020 [1]. It is caused by *Plasmodium* parasites, the most prevalent being *Plasmodium falciparum* and *Plasmodium vivax.* Seasonal transmission of *Plasmodium* parasites, that is, low transmission during the dry season and higher transmission during the wet season, is common in malaria endemic regions [2, 3]. During the dry season, the transmission rate is very low, in some areas it is near zero [4, 5]. In malaria endemic regions, adults are often asymptomatic (used here to mean infection with *Plasmodium* parasites, which is sub-acute, with the patient not reporting any malaria-related symptoms) but harbour low parasite loads [2, 6–9]. These sub-patent malaria infections have also been found during the dry season [6, 7, 9, 10] and have led to the conclusion that humans provide a reservoir for parasites during the dry season [3, 7, 11]. Recently, Andrade et al*.* have shown that parasites survive at low levels in certain individuals and carriers have higher humoral immunity and protection against clinical malaria than non-carriers [10], as was also shown by Portugal et al*.* [4]. At the same study site 12–30% of individuals carried *P. falciparum* parasites at the end of the dry season in different years [4, 10]. This subset of individuals will likely contribute to the infection of the vector population at the commencement of the wet season and thereby re-seed the next transmission season. This is supported by studies showing that the duration of some infections are commonly longer than 6 months [12–15] and other studies showing that the genetic diversity of parasites is preserved in the dry season [4, 7, 10]. However, it remains unclear whether this subset of individuals who carry infection is a random subset of the individuals exposed to infection in the previous season or if they have some other factors contributing to their propensity to carry parasites and if some evidence exists for the latter. Thus, this study aims to characterize these dry season carriers.

Age appears to be an important correlate of both carriage [16] and contribution to the infectious reservoir [17, 18]. In high transmission regions, age is both a determinant of the parasite density distribution [6] and cumulative past exposure [19]. Some evidence exists to suggest that older children and adults have longer lasting infections, and that immunity increases with age [20–27]. Previous modelling work has hypothesized a mechanism by which increased immunity as a result of age and exposure may drive longer durations of infection [21, 22, 25–27]. This may occur due to antibody-mediated cross-reactive immunity that controls diverse *P. falciparum* strains, but it may also be due to a more general physiological response such as hypersplenism [25–27] or changes in the activation of endothelial cells and reduced sequestration, which lead to overall slower growing parasitaemia [10, 28, 29]. These different types of immunity, cross-reactive and strain-specific immunity, have been incorporated in previous models of malaria infections [25, 30]. Regardless of the mechanism, modelling has suggested that if exposure to infection induces immunity, longer-lived infections will occur as an individual’s exposure increases. In addition to age, individuals may also vary in their degree of exposure [16, 31]. Factors that may influence an individual’s exposure are variable and may include behavioral, social, entomological and biological factors such as place of residence, occupation, sleeping outside, bed net use, local use of insecticides and age. For this reason, some may acquire a level of immunity sufficient for long-lived infections earlier in life than others. Therefore, in the following, the role of exposure to infection in determining parasite carriage across the dry season is explored.

First, a mathematical model of repeated infections within an individual is used to assess the relationship between exposure, age, and parasite carriage and explore the stochastic variation in an individual’s propensity to carry infection. To explore whether this stochasticity is sufficient to explain why some individuals carry parasites over the dry season, the model simulations are compared with cohort data from Mali [4, 32]. In this cohort data, parasite carriage is not purely stochastic and is in fact more likely in individuals who are most at risk of infection. The highly exposed individuals who carry parasites over the dry season, if they are bitten more frequently, are likely to re-seed infection at the beginning of the next wet season and during the wet-season are predicted to cause a 2.6-fold faster infection of the mosquito population than if carriage of infection over the dry-season is from a random subset of individuals. This suggests that dry-season carriers may play a role as “super-spreaders” in seasonal transmission settings.

## Methods

### Model

The model contains stochastic infectious mosquito bites and deterministic within-host dynamics [25]. In the model, infectious mosquito bites occur randomly. The infectious biting rate was assumed to be piecewise constant with no infectious bites during the dry season and a constant infectious biting rate throughout the malaria transmission season from July through December. Parasite diversity is incorporated by assuming that with every infectious mosquito bite a new parasite strain is inoculated. After the infectious bite and the liver stage of infection (which was modelled as a fixed time delay between the infectious bite and the beginning of the blood-stage), a fixed number of parasites are released into the blood, i.e., the initial parasite concentration depends on the blood volume). Each infectious bite induces strain specific and cross-reactive immunity. The within-host dynamics of infections are deterministic and described by the following model:

$$\begin{array}{*{20}l} {\frac{{dP_{i} }}{dt} = \left( {\frac{\ln \left( r \right)}{2} - C - S_{i} } \right) P_{i} ,} \hfill \\ {\frac{{dS_{i} }}{dt} = \alpha P_{i} ,} \hfill \\ {\frac{dC}{{dt}} = \gamma \left( {\mathop \sum \limits_{j = 1}^{\infty } P_{j} } \right) {-} \delta C,} \hfill \\ \end{array}$$

where ${P}_{i}$, $i=\mathrm{1,2},3,\dots$, is the parasite concentration of the $i$ th strain (in parasites/µl), ${S}_{i}$ is the strain specific immunity, and $C$ is cross-reactive immunity. Thus, parasites grow at a certain rate ($r$ is the parasite multiplication rate, hence the growth rate is ${\text{ln}}(r)/2$), this growth is slowed by cross-reactive and strain specific immunity. If the parasite concentration falls below the clearance threshold (${Z}_{P}$), then the parasite is cleared. Strain specific immunity increases at rate $\alpha$ proportional to the parasite concentration and does not decrease in the presence of the parasite. Cross-reactive immunity increases at rate $\gamma$ proportional to the overall parasite concentration and decreases linearly. For the interpretation of the parameters and the values used for the simulation see Additional file 1: Table S1.

### Model simulations

The model was simulated in MATLAB [33] using the parameter values in Additional file 1: Table S1. Individuals have random dates of birth, random age at the beginning of the wet season (uniformly distributed between 3 months and 12 years), and a randomly chosen Force Of Infection (FOI) (uniformly distributed between 0.04 and 0.004 infectious bites per day during the wet season). If the parasite concentration of any strain decreased below the threshold ${Z}_{P}$, then the parasite concentration was set to zero (without this threshold the parasite would never be cleared completely). Furthermore, the strain specific immunity of a strain that was cleared was also set to zero as it does not contribute to the within-host dynamics anymore (every infection is an infection with a new strain, the same strain is never inoculated again). At the end of the dry season, all individuals were classified based on their overall parasite concentration and depending on the limit of detection of a Rapid Diagnostic Test (RDT) or Polymerase Chain Reaction (PCR) as either RDT^+^, RDT^+^PCR^−^, or PCR^−^ with limits of detection 100 parasites per μL and 1 parasite per μL respectively. Treatment of RDT^+^ individuals was simulated by setting the parasite concentration to zero. All individuals were then simulated for 1 year. For more details on the model simulations see the Supplementary Information.

### Data from Mali

The data was collected in a longitudinal study conducted in Mali in 2012. This data set and the details of this study were previously described [4, 32]. Individuals aged 3 months–12 years were enrolled in May 2012. In the study area in Mali, malaria is seasonal with no or only very little transmission during the dry season from January through June and malaria transmission during the wet season from July through December [4]. At enrolment, i.e. at the end of the dry season, study participants were tested for *P. falciparum* parasites using a Rapid Diagnostic Test (RDT) and Polymerase Chain Reaction (PCR). Out of the 579 study participants, 100 (17.3%) had positive RDT results and were treated with anti-malarials, 55 (9.5%) had negative RDT results, but positive PCR results, and 424 (73.2%) had negative RDT and PCR results (Fig. 6). Overall, 26.8% of children were found to carry parasites at enrolment. There is a gap of 2 months between enrolment (in May) and the first follow-up visit (in July) where active follow-up visits were not possible due to limited access to the study area. From July 2012 to the end of December 2012, there were 12 fortnightly follow-ups with PCR test for RDT positive (treated) and PCR negative study participants. Additionally, episodes of clinical malaria were detected by weekly active clinical surveillance visits and self-referral. The ethics committee of the Faculty of Medicine, Pharmacy and Dentistry at the University of Sciences, Techniques and Technology of Bamako, and the Institutional Review Board of NIAID NIH approved the study (ClinicalTrials.gov NCT01322581). Written, informed consent was obtained from the parents or guardians of participating children or from adult participants.

### Data analysis and statistical information

The software R (version 3.6.0) [34] was used to analyse the data. The data from Mali was analysed using the package dplyr (version 0.8.3) [35] and survival analysis with the packages survival (version 2.38) [36, 37] and survminer (version 0.4.5) [38]. In boxplots, the length of the whiskers is the minimum of 1.5 times the interquartile range and the distance from the box to the largest or lowest (for upper and lower whiskers respectively) data value. Survival curves were compared with the log-rank test and different groups were compared with one-sided Wilcoxon rank-sum tests. Duplicate data for the same individual (that is, repeated entries for the same subject ID) were excluded. If there were inconsistencies in the follow-up dates (e.g., third visit before the second visit), then only the follow-ups before the first inconsistent date were considered. For time from first follow-up to PCR^+^, individuals with a positive PCR result at the first follow-up visit were excluded and maximally 20 days between known PCR results were allowed. If the time between two known PCR results is more than 20 days, then this case was considered as right censored before this gap. Additionally, the analysis was repeated with the time from the first follow-up instead of time from enrolment to first positive due to the gap of approximately 60 days between enrolment and the first follow-up with a PCR test (the analysis yielded the same results in both cases, see Supplementary results). For time to clinical malaria, if an individual had no observed episode of clinical malaria, then this individual was considered as right censored at the date of the last follow-up.

## Results

### Simulation demonstrates a random subset of individuals can carry infection over the dry season

**Fig. 1:**
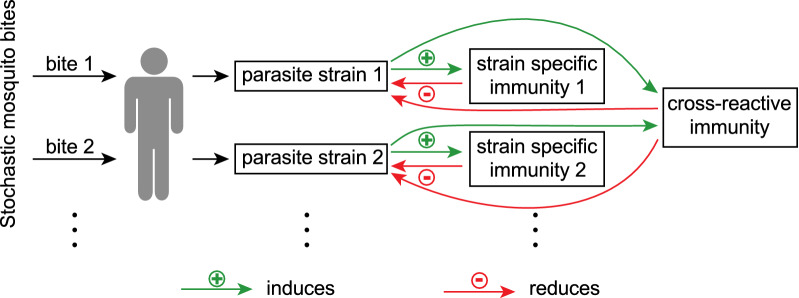
Schematic of the mathematical model used to simulate the malaria life history of individuals. Individuals receive stochastic and seasonal infectious mosquito bites. Each infectious bite inoculates a new parasite strain. The growth of a parasite strain induces strain specific immunity to that strain and cross-reactive immunity to all strains. Immunity reduces the growth rate of either a specific strain (strain specific immunity) or all strains (cross-reactive immunity). If the parasite concentration is low, cross-reactive immunity decreases whereas strain specific immunity only decreases when the corresponding parasite strain is cleared. More information on the model can be found in the Methods and the supplementary methods. Representative simulations of individuals with different exposure to infectious mosquito bites are shown in Fig. 2, Additional file 1: Figures S1 and S2

**Fig. 2:**
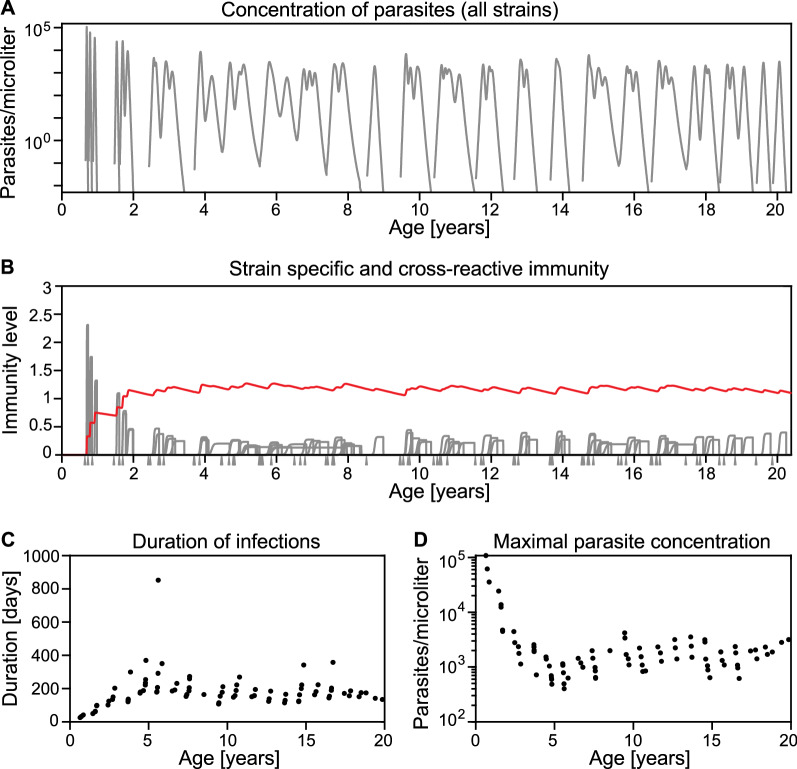
Simulation of one individual from birth to age 20. This simulation shows an individual with a mean biting rate of 0.022 infectious bites per day (all other parameters as in Additional file 1: Table S1). **A** Overall parasite concentration, i.e., the concentration of all parasite strains together, with age. **B** Strain specific immunity (grey lines) and cross-reactive immunity (red line) vs age. Cross-reactive immunity may be antibody-mediated cross-reactive immunity or a more cross-reactive physiological response. The grey triangles indicate infectious mosquito bites. Note that strain specific immunity is only plotted for the time interval the respective strain is present in the blood. **C** Duration of infections with age. The duration of an infection is the time from the beginning of the blood-stage to clearance of the parasite strain. **D** Peak parasite concentration of each infection that occurs in the first 20 years of an individual’s life from the simulation, i.e., the maximal parasite concentration for each infection. As the individual generates more cross-reactive immunity with age and exposure, the peak parasite concentrations experienced by that individual decline. For simulations of individuals with a different exposure, see Additional file 1: Figures S1 and S2

The influence of exposure and age on the probability of an individual carrying infection over the dry season was explored using a simulation model based on a model by Pinkevych et al*.* [25] (Fig. 1and Methods). This model simulates an individual’s exposure to repeated infections. Infectious bites are stochastic and induce both strain specific immune responses and cross-reactive immune responses. Each infectious bite is assumed to be a different strain, and cross-reactive immunity decays with a 5-year half-life. The first 20 years of individuals' simulated infection histories, under different Forces Of Infection (FOIs), are shown in Fig. 2 (see also Additional file 1: Figures S1 and S2). After an infectious mosquito bite with a particular strain, the parasite concentration increases which induces an increase in strain specific immunity and a slower increase in cross-reactive immunity (Fig. 2A, B). High parasite loads in early childhood infection induce large numbers of antibodies in the model. Thus, the less well controlled an infection is, the higher the peak of strain specific immune response (Fig. 2). This result follows from the model assumption that antibody production is correlated with pathogen load. It follows that the first infections are predominantly controlled by strain specific immunity, but as the individual experiences repeated infections, cross-reactive immunity has a much greater contribution to the control of infections (Fig. 2B). Higher levels of cross-reactive immunity led to a decrease in the maximal parasite concentration, and in turn lower peak strain specific immune responses. A property that emerges from the model is that as cross-reactive immunity increases, the lower peak in parasite concentration and strain specific immunity lead to a longer duration of each infection (Fig. 2C, D).

**Fig. 3:**
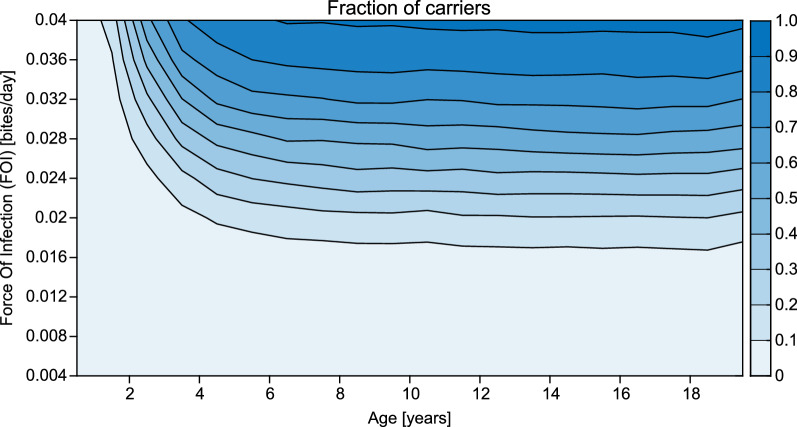
The fraction of simulated individuals who carry parasites over the dry season by FOI and age. For each of ten different FOIs between 0.004 and 0.04 infectious bites per day during the wet season, 10,000 individuals were simulated from birth to age 20. The different shaded areas indicate which fraction of the simulated individuals has carried parasites through the dry season at the respective age. Parasite carriage is defined as PCR detectable parasite concentrations in an individual on the last day of the dry season. Individuals with a higher exposure carry parasites at a younger age, while some individuals who are less exposed never carry parasites over the dry season before they reach age 20 (see also Additional file 1: Figures S3 and S4)

Simulations were repeated 10,000 times for each FOI tested. These results revealed that the age of an individual first carrying parasites over the dry season decreases nonlinearly with increasing FOI (Additional file 1: Figures S3 and S4). Moreover, the probability of an individual carrying parasites is higher with higher FOIs (Fig. 3). These simulations reveal that for a given exposure and age, there is a certain chance an individual will carry parasites over the dry season, and a chance they will not. Next, it was explored whether any factor predicted whether an individual would carry parasites over a dry season.

**Fig. 4:**
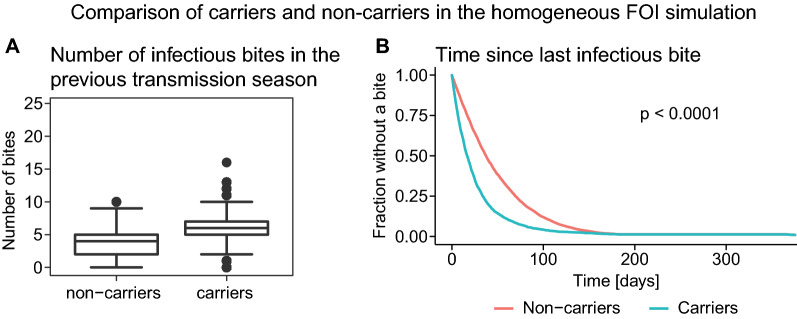
Simulation of individuals with a FOI of 0.024 infectious bites per day. For this simulation, 10,000 individuals with the same exposure were simulated to an age uniformly drawn between 0 and 20 years. Individuals were classified as carriers if they had a parasite concentration above the limit of detection for a Rapid Diagnostic Test (RDT) at the end of the dry season. **A** The number of infectious bites that individuals received in the transmission season before they were classified as carriers or non-carriers is significantly higher for carriers than for non-carriers (Wilcoxon rank-sum test with p < 0.0001). **B** The time from the last infectious bite to the end of the previous transmission season is significantly shorter for carriers than for non-carriers (log-rank test with p < 0.0001). For a comparison of the number of infectious bites in the previous season and the time since the last infectious bite between carriers and non-carriers for other FOIs see Additional file 1: Figures S5 and S6

For a given FOI, individuals who carried parasites over a dry season had experienced significantly more infectious bites and a more recent infectious bite in the previous wet season than individuals who did not carry parasites (Fig. 4, Additional file 1: Figures S5 and S6). Together these modelling results predict that a history of high exposure to infection may increase the risk of carriage over the dry season and that parasite carriage may be purely random. That is, the same individual may or may not carry parasites over a dry season depending on their recent infection history.

### Time to next infection distinguishes stochastic and exposure-mediated dry season carriage

**Fig. 5:**
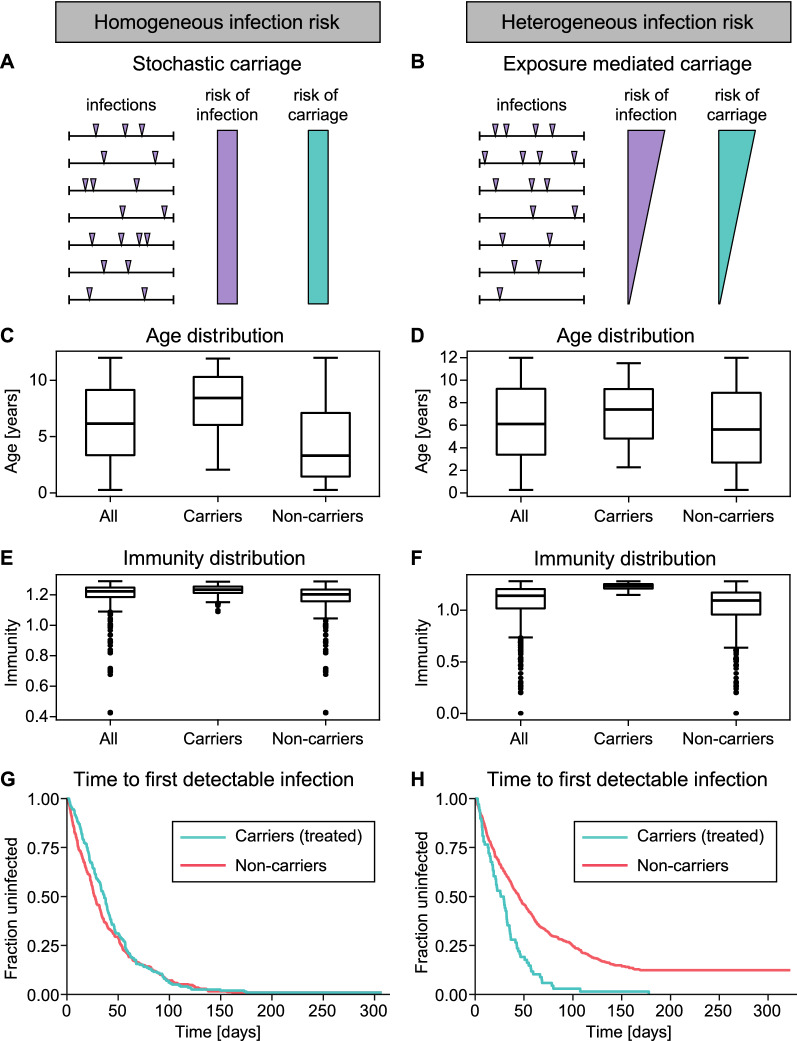
Stochastic or exposure mediated carriage of parasites over the dry season in the homogeneous or heterogeneous exposure settings, respectively. **A** If parasite carriage over the dry season is stochastic in a homogeneous population, then all individuals have a similar risk of infection and risk of carriage. **B** If parasite carriage is exposure mediated, then the individuals with a higher risk of infection also have a higher risk of parasite carriage over the dry season. In this scenario, there is still stochasticity in carriage but the risk of carriage that correlates with the risk of infection. **C-H** 1,000 simulated individuals aged between 3 months and 12 years with either homogeneous or heterogeneous infection risk during the wet season. Individuals were classified as carriers or non-carriers depending on whether they have a parasite concentration above the limit of detection for a Rapid Diagnostic Test (RDT) at the end of the dry season. The simulation included treatment of carriers at the end of the dry season. Parameter values for this simulation can be found in Additional file 1: Table S1. The biting rate for the homogeneous infection risk simulation is 0.024 infectious bites per day (for simulations of a homogeneous population with other biting rates see Additional file 1: Table S2 and Figure S7). **C, D** In both the homogeneous and heterogeneous simulations, carriers were significantly older than non-carriers (Wilcoxon rank-sum test with p < 0.0001 and 0.0005, respectively). **E, F** Carriers have a significantly higher cross-reactive immunity than non-carriers in both the homogeneous and heterogeneous infection risk simulation (Wilcoxon rank-sum test with p < 0.0001 in both cases). **G, H** Time from the first follow-up to infection (by PCR, i.e., the overall parasite concentration exceeds the limit of detection for PCR). In the simulation with homogeneous risk of infection, there is no significant difference in the time to the next infection between carriers and non-carriers (log-rank test with p-value 0.2). In the heterogeneous infection risk simulation, carriers had a significantly higher infection risk compared to non-carriers (log-rank test with p < 0.0001)

The above modelling demonstrated that purely stochastic differences are a possible reason why a fraction of individuals with similar immunity and history of exposure may carry parasites over the dry season while others do not, and there may be no distinctive characteristics of the carrier population. However, since the probability of dry season parasite carriage increases with the FOI (Fig. 3), it is also possible that dry season carriers are not just a random subset of individuals in a community but that they are the most frequently infected. Two possible scenarios are simulated: (1) a community of individuals exposed to a homogenous FOI, and (2) a community of individuals exposed to a heterogenous FOI. In these two scenarios, the subset of individuals who carried parasites over the dry season is identified and characterized. These two scenarios show some features that are consistent. In both the homogeneous and the heterogeneous exposure settings, carriers are significantly older and more immune than non-carriers (Fig. 5C–F, Additional file 1: Table S2, Figures S8 and S11). The difference between the homogeneous and the heterogeneous simulation is that in the latter, carriers are more exposed than non-carriers (Fig. 5H, Additional file 1: Figures S8 and S11) while in the homogeneous case there is no difference in the exposure of carriers and non-carriers (Additional file 1: Table S2). Interestingly, despite the systematically higher risk of infection between carriers and non-carriers in the heterogeneous population model, there is also stochasticity in the heterogeneous case—while older and more exposed individuals are more likely to be carriers, some younger or less exposed individuals may also be carriers (Additional file 1: Figure S9) and it is not necessarily the same individuals that carry parasites over different dry seasons (Additional file 1: Figure S10). Together, this modelling predicts that in a heterogenous population a subset of individuals with higher risk of infection are more likely to carry parasites over the dry season.

### Heterogeneity in infection risk in Mali cohort indicates that high risk of infection leads to high risk of dry season carriage

**Fig. 6:**
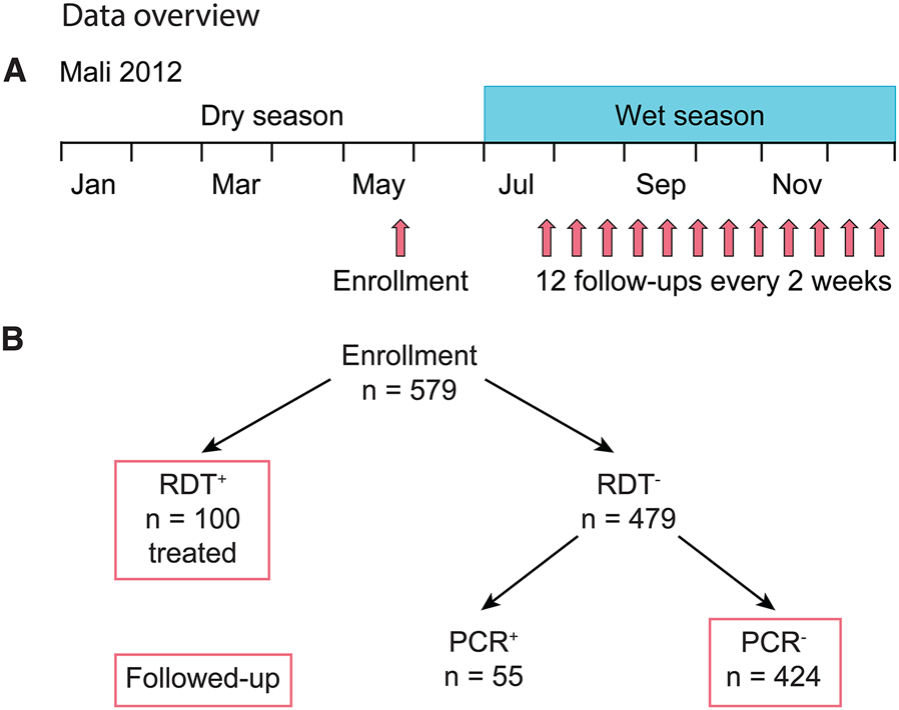
Data overview. **A** Children were enrolled in May 2012 at the end of the dry season (January–June). During the wet season, when malaria transmission takes place, enrolled children were visited every 14 days to the end of the year. Note that due to the political situation in Mali in 2012, there is a gap of 2 months between enrolment which took place in May 2012 and the first follow-up visit in July 2012. In order to take this gap in the data into account, the data analysis was performed both with enrolment and the first follow-up visit as first visit date (see Supplementary information) with the same results in both cases. **B** At enrolment, children were tested with RDTs. Those with positive test results were treated and those with negative results were additionally tested with PCR. Only RDT positive and RDT and PCR negative children were followed-up regularly

**Fig. 7:**
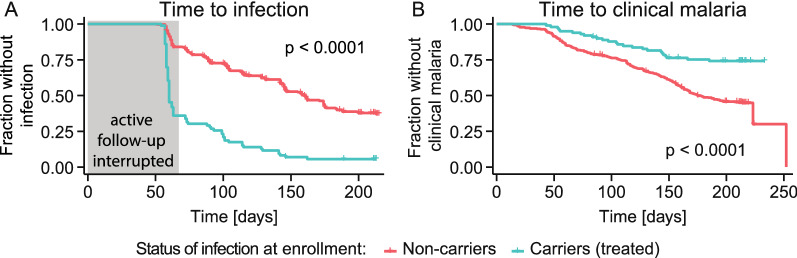
Time to infection and time to clinical malaria. **A** Time from enrolment to first detection of parasites with PCR for treated carriers and non-carriers. Note that active follow-up was interrupted for about 60 days between enrolment in May and the first follow-up visit in July due to limited access (see Methods and Supplementary information). Carriers have a higher risk of infection compared to non-carriers (log-rank test with p < 0.0001). **B** Time from enrolment to clinical malaria for carriers and non-carriers. This plot is a reproduction of data published by Portugal et al. [4] who have shown that carriers are more immune to clinical malaria than non-carriers. Non-carriers have a significantly higher risk of clinical malaria than carriers (log-rank test with p < 0.0001). For the time to infection and clinical malaria from the first follow-up visit instead of enrolment, see Additional file 1: Figure S16

The above analysis showed that a systematic difference of higher exposure or random differences may explain why some individuals carry infections over the dry season and others do not. Here, this model prediction is investigated in data from a seasonal malaria setting [4, 32]. In particular, it is considered whether there is any evidence that dry season carries are systematically more highly exposed individuals as previously hypothesized [4, 10]. In this cohort study (previously published by [32 and 4]), children aged 3 months–12 years were tested for *P. falciparum* infection at the end of the dry season (using RDT and PCR) and all individuals who were RDT positive were treated (Fig. 6). Children were then followed up for infection (by PCR) fortnightly to the end of the year. Individuals who were positive for infection with a positive RDT result at the end of the dry season are assumed to be dry season carriers, while individuals without parasites (PCR and RDT negative) are assumed to be non-carriers (see Methods for more details on the data). These studies previously reported that carriers are more immune to clinical malaria than non-carriers [4, 10], antibody levels decline similarly in carriers and non-carriers [10], older children are more likely to be carriers [4], and risk of clinical malaria decreases with age despite no age-related differences in the infection risk [32]. Under both modelling scenarios older individuals with higher cross-reactive immunity were more likely to carry parasites over the dry seasons. Consistent with this in the cohort study, it has previously been shown that carriers were significantly older than non-carriers (data reproduced, Additional file 1: Figure S12) [4, 10], and carriers had a lower risk of clinical malaria than non-carriers (data reproduced here, Fig. 7B, p < 0.0001) [4, 39, 40]. Thus, consistent with the model, these previous studies indicate that carriers have a greater immunity to clinical malaria than non-carriers [4, 39, 40].

Finally, to determine whether carriers had a systematically higher FOI, the time to infection (by PCR) for carriers and non-carriers was compared in a novel analysis of the Mali data. Using both a log-rank test and Cox Proportional Hazards model to compare the time to infection data for carrier and non-carriers, the data shows that carriers had a significantly higher risk of infection compared to non-carriers, even after adjusting for age (Fig. 7A, log-rank test with p < 0.0001, see also Additional file 1: Figures S13 and S16, Cox model with p < 0.0001 for both age and parasite carriage, see Additional file 1: Tables S3, S4, S5 and Figures S15, S17, S18). Thus, those who carry parasites at the end of the dry season have a higher risk of infection at the beginning of the wet season compared with non-carriers. This indicates that dry season carriage is not a purely random process and that carriers have a systemically higher exposure to infection.

### Heterogeneity in infection risk predicted to facilitate the fast spread of malaria at the beginning of the wet season

**Fig. 8:**
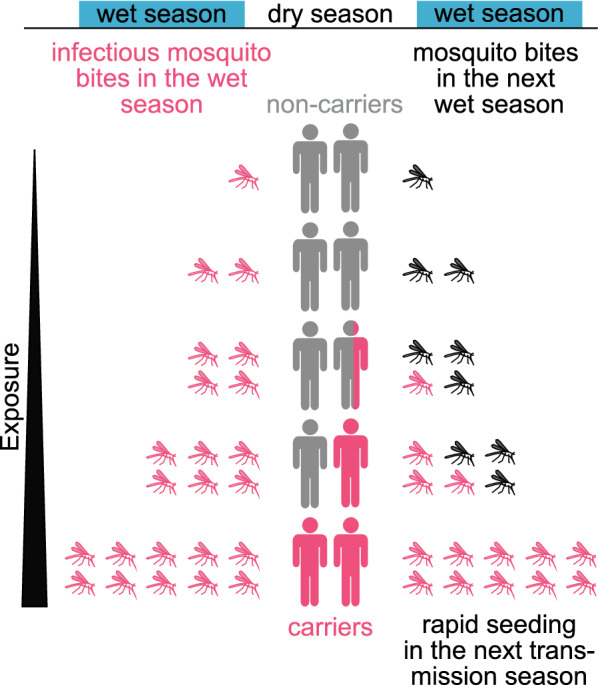
Heterogeneity in exposure may facilitates the fast spread of parasites in the next transmission season. The most exposed part of the population is most likely to carry parasites over the dry season (red individuals). In the next wet season, these most-exposed individuals receive more infectious bites than non-carriers leading to the rapid infection of the mosquito population (red mosquitoes)

From the dataset from Mali, it is evident that carriers have a 3.8-times higher risk of infection in the ensuing wet season compared with non-carriers (Additional file 1: Figure S13). Carriers only account for 17.3% of the population at the end of the dry season in this data set (Fig. 6), and hence this subgroup of highly-exposed carriers may allow for rapid transmission in the subsequent wet-season [31]. If mosquitos feed on dry season carriers 3.8 times as much as on non-carriers (Additional file 1: Figure S13), then the 17.3% of individuals who are carriers are estimated to have received 44.1% of all mosquito bites at the start of the wet season, due to their higher exposure (see Supplementary methods). In contrast, if all individuals were randomly received bites from the mosquito population, the dry season carrier population is expected to only receive 17.3% of all mosquito bites. In a simple model of the spread of parasites in the mosquito population, the time until half of all the mosquito population is infected is 60.8% shorter in the case that dry season carriers are 3.8-times more likely to be bitten, compared with a scenario where biting is homogeneous across the population (Fig. 8). Thus, modelling predicts that due to heterogeneity in infection risk, parasites spread 2.6 times faster in the mosquito population at the beginning of the wet season as they would if carriers and non-carriers had the same risk of infection.

## Discussion

Consistent with the proposed model where higher parasite exposure leads to an increase in duration of infections via cross-reactive host immunity, favoring dry season persistence of infections, data from a Malian cohort shows that dry season carriers have a higher risk of infection, despite their known increased protection from clinical malaria [4, 39, 40]. These findings are also consistent with other studies that have found that in high transmission regions, immunity increases with age and the duration of infections increases with immunity [19–23]. Other studies have identified exposure to infection as an important factor influencing immunity and asymptomatic parasitaemia in children [24, 41]. Together these studies suggest that an individual’s history of exposure to infection is a strong determinant of the duration of their infections and parasite carriage over the dry season (Additional file 1: Figure S14). Of note, the modelling study presented here has attempted to draw together multiple observations from the literature, as well as the novel data presented here, including the demographic characteristics, higher exposure and lower incidence of clinical malaria in dry season carriers, and provide a unified explanation of these observations, that is, that exposure mediated immunity driving longer infections may facilitate carriage across the dry season.

The observation that dry season carriers had a shorter time to infection in the next wet season indicates heterogeneity in exposure to infectious bites within the cohort studied. Importantly, it has not been possible to dissect the origins of this heterogeneity in exposure. Heterogeneity may arise from socio-economic and behavioral differences [42], such as dwelling, micro-geographical heterogeneity in the mosquito density [43], bed net use or insecticide use within the home. However, more fundamental host differences may also contribute to this heterogeneity, for example, blood cell traits associated with reduced risk of infection, and host differences modulating risk of a mosquito blood meals [44].

For each level of exposure and age, there is a certain risk of persistent infections that last the entire dry season. However, there is also stochasticity in parasite carriage. It is possible that there is a “sweet spot” of immunity for parasite carriage that depends on the regularity and temporal pattern of immunity boosting infections. Indeed, it has been observed that older children (aged 5–15 years) rather than young children (less than 5 years old) or teenagers and adults (at least 16 years old) contribute disproportionately to the infectious reservoir [45]. Thus, parasite carriage may be a transient phenotype with older children being the most likely carriers since they are most likely to have had a certain level of immunity-boosting cumulative exposure without being sufficiently immune to clear all parasites during the dry season.

It has been previously observed that both super-spreading and sub-patent parasite loads play an important role in malaria transmission, in particular in low-transmission settings with malaria transmission throughout the year [31, 46]. This analysis indicates that the same is likely to be true in seasonal malaria transmission settings [45]. Carriers have a shorter time to the next infection at the beginning transmission season as well as from the first follow-up visit (about 1 month into the transmission season, see Additional file 1: Figure S16), indicating a higher level of exposure of carriers to mosquito feeding, at least early in the transmission season. However, biting rate is not the only factor determining transmission, and an individual’s potential to infect a mosquito is likely to depend on the number of gametocytes the individual carries and their infectiousness, which was not confirmed. Although no direct data on the rate at which carriers contribute to new infections of mosquitoes is available, this modelling suggests that heterogeneity in the biting rate with carriers being more exposed than non-carriers at the start of the transmission season may facilitate the fast spread of parasites at the beginning of the transmission season (Fig. 8). Mosquitoes preferentially biting infected individuals [47, 48] may further aid in the fast spread of malaria at the beginning of the wet season. To the extent that it is possible to identify these individuals, elimination efforts must target the most highly exposed individuals, when aiming to interrupt the emergence of parasites in the wet season.

A number of limitations exist in this study. In particular, the modelling of host strain specific and cross-reactive immunity makes a number of simplifying assumptions (Supplementary methods), but promisingly yields similar predictions of the impact of exposure driven immunity on duration of infection as has been reported by other more detailed models [12, 13]. Further, the cohort used in this study only includes children, and thus may contain some bias since adults have been suggested to be the primary parasite reservoir [17, 18]. This is important as it cannot be excluded that the same properties of carriage linked to higher exposure would be evident in the adult population. Additionally, it is worth noting that the model presented here, analysing exposure within an individual, is not a full model of transmission and only a simplified illustrative model of infection into a hypothetical mosquito population in a wet season was considered. More advanced transmission models could be leveraged to explore the full impacts of a link between carriage and exposure in the population in future work. More comprehensive models would likely indicate that the time to 50% of the mosquito population is infected is a threshold that may never be reached [49], none-the-less the simplified model presented here provides a useful tool for comparison of the different feeding scenarios. Finally, it cannot be excluded that other mosquito-linked factors may be important drivers of parasite survival over the dry season, such as dry season hot-spots of transmission, allowing parasite sporogonic stages to survive [50], and mosquito migration [51].

If only a fraction of the population carries parasites over the dry season and re-seeds the following transmission season, then the question arises whether this is an advantage for malaria elimination efforts. On the one hand, fewer individuals need to be treated before the wet season as opposed to a larger proportion of the population during the wet season [16]. On the other hand, the difficulty lies in identifying these carriers [52]. This modelling indicates that carriage of parasites over the dry season relates to the risk of infection, but there is also stochasticity in parasite carriage since for any given FOI there is still a proportion of individuals who will not carry parasites over the dry season. Mass Drug Administration (MDA) could be a possible solution to deal with this difficulty. However, if carriers are missed in MDA programmes, the effect of MDA may be short-lived due to the fast spread of parasites at the beginning of the wet season—as was suggested by previous modelling work [53, 54]. Repeated rounds of MDA—timed to take optimal advantage of the seasonality—in combination with vector-control may still be necessary to reduce the disease burden [16, 53–56].

## Conclusions

Carriage of malaria parasites over the months-long dry season in Mali is most likely in the older, more exposed and more immune children. A within host model of repeated infections indicates that high levels of exposure lead to high levels of immunity which in turn can lead to long-lasting infections. These highly exposed children who carry parasites over the dry season may also act as super-spreaders facilitating the fast spread of parasites at the beginning of the next transmission season.

## Supplementary Information

**Additional file 1: Table S1.** File containing, supplementary tables (S1-S5), supplementry figures (S1 - S18) and supplementary methods andresults.

## Data Availability

The data and code are avaliable at https://github.com/InfectionAnalytics/Malaria-Dry-Season-Carriage.

## Abbreviations

FOI: Force of infection
RDT: Rapid diagnostic test
PCR: Polymerase chain reaction
MDA: Mass drug administration
